# Impacts on Thrombus and Chordae Willisii During Mechanical Thrombectomy in the Superior Sagittal Sinus

**DOI:** 10.3389/fneur.2021.639018

**Published:** 2021-03-04

**Authors:** Yuanliang Ye, Jiuyang Ding, Shoutang Liu, Shaoming Huang, Zhu Li, Jianqing Yang, Jiang Huang

**Affiliations:** ^1^Department of Neurosurgery, Liuzhou People's Hospital, Liuzhou, China; ^2^School of Forensic Medicine, Guizhou Medical University, Guiyang, China; ^3^Department of Anatomy, Guangxi Medical University, Nanning, China; ^4^Department of General Surgery, Liuzhou People's Hospital, Liuzhou, China

**Keywords:** cerebral venous thrombosis, mechanical thrombectomy, chordae willisii, embolism, intervention

## Abstract

The anatomical structures of the superior sagittal sinus (SSS) are usually damaged during mechanical thrombectomy (MT), and MT procedure could lead to new thrombosis in the sinuses. However, the mechanism remains unclear. We aimed to investigate the risks of embolism and assess the damage to chordae willisii (CW)-associated MT using a stent passing across the thrombus. A contrast-enhanced *in vitro* model was used to mimick MT in the SSS. The thrombus was removed with a stent. The emboli generated during the procedure were collected and measured. The residual thrombus area after the MT was measured by J Image software. The damage of CW was evaluated by an endoscope. Three procedural experiments were carried out on each cadaveric sample. The average numbers of visible emboli particles in experiments 1, 2, and 3 were 11.17 ± 2.17, 9.00 ± 2.07, and 5.00 ± 2.96, respectively. The number of large size particles produced by experiment 1 was significantly higher than that of the other experiments. The thrombus area measured after experiment 3 was larger than that of experiments 1 and 2. The number of minor damage cases to CW was 55 (90.16%), and there were six serious damage cases (9.84%). The use of stent resulted in no significant increase in damage to CW after the three experimental procedures. A large amount of thrombi particles was produced during MT, and multiple MT procedures on the same sample can increase residual thrombus area. Moreover, the stent caused minor damages to the CW in SSS.

## Introduction

Venous thrombosis can affect all veins in the body including the cerebral venous system leading to cerebral venous thrombosis (CVT) (1). CVT accounts for 0.5% of all strokes with an annual incidence ranging from three to four cases per million among the general population and up to seven cases per million among the youths (2, 3) with a fatality rate of 6–10% (4, 5). Timely institution of systemic anticoagulation is recommended as the first-line treatment based on efficacy demonstrated in clinical trials (6–9). However, 10–20% of patients either deteriorate despite medical treatment or present with symptoms of intracranial hypertension. Direct intra-sinus infusion of thrombolytic agents may be an effective treatment (10), but this approach may require several days to re-establish anterograde venous outflow in the targeted sinus(es) and may increase the risk of hemorrhagic complications, especially in patients with preexisting hemorrhagic venous infarction. Mechanical evacuation of thrombus and quick restoration of venous flow can reduce the total volume of venous thrombus burden and prevent stasis and further propagation of thrombus (11). Mechanical thrombectomy (MT) is reasonably safe and effective (12). It increases the surface area of the thrombus exposed to intra-sinus thrombolysis (13–15).

The complications during the MT included perforation of venous sinuses, vessel dissection, and emboli formation (16–18). The emboli burden was related to the disruption of the thrombus (19, 20), which produces clot fragments that flow distally from the dura sinuses into the pulmonary vasculature (21). Moreover, The MT may cause iatrogenic damage to the chordae willisii (CW), which exists in the SSS and, upon damage, would expose its collagen fibers and cause thrombosis (22). In the present study, we created an experimental model with thrombus formation in SSS and mimicked a mechanical thrombectomy (MT) process. We investigated the risks of emboli formation via a stent manipulation of the thrombus and assessed the damage to CW by the stent via an endoscope.

## Materials and Methods

A total of 16 cadaveric heads, six males and 10 females, were obtained from autopsies performed at the Department of Anatomy, Guangxi Medical University. The consent of usage form was signed by the donor or the relatives of the donors who were all above age 18. The mean age of these specimens was 58 ± 11.48 years old (range 38–75). This study was approved by the ethics committee of Guangxi Medical University (IRB approval ID No. KY-E-01-01). All cadaveric heads were fixed in 10% formalin solution for 4–6 weeks. The exclusion criteria were as follows: (1) craniocerebral trauma, (2) neurological disease, and (3) disease affecting the dura sinuses.

### Preparation of Experimental Thrombosis Model in Superior Sagittal Sinus

Briefly, 10 ml of swine whole blood was mixed with 25 IU of bovine thrombin (Dade Behring, Newark, Delaware). One gram of barium sulfate was mixed with the blood component for 10 s, and the mixture was kept in a silicone tube for 60 min at room temperature (23–25). After the incubation period, the created artificial thrombus was removed from the silicone tube and cut to a length of 20 mm.

**Figure 1:**
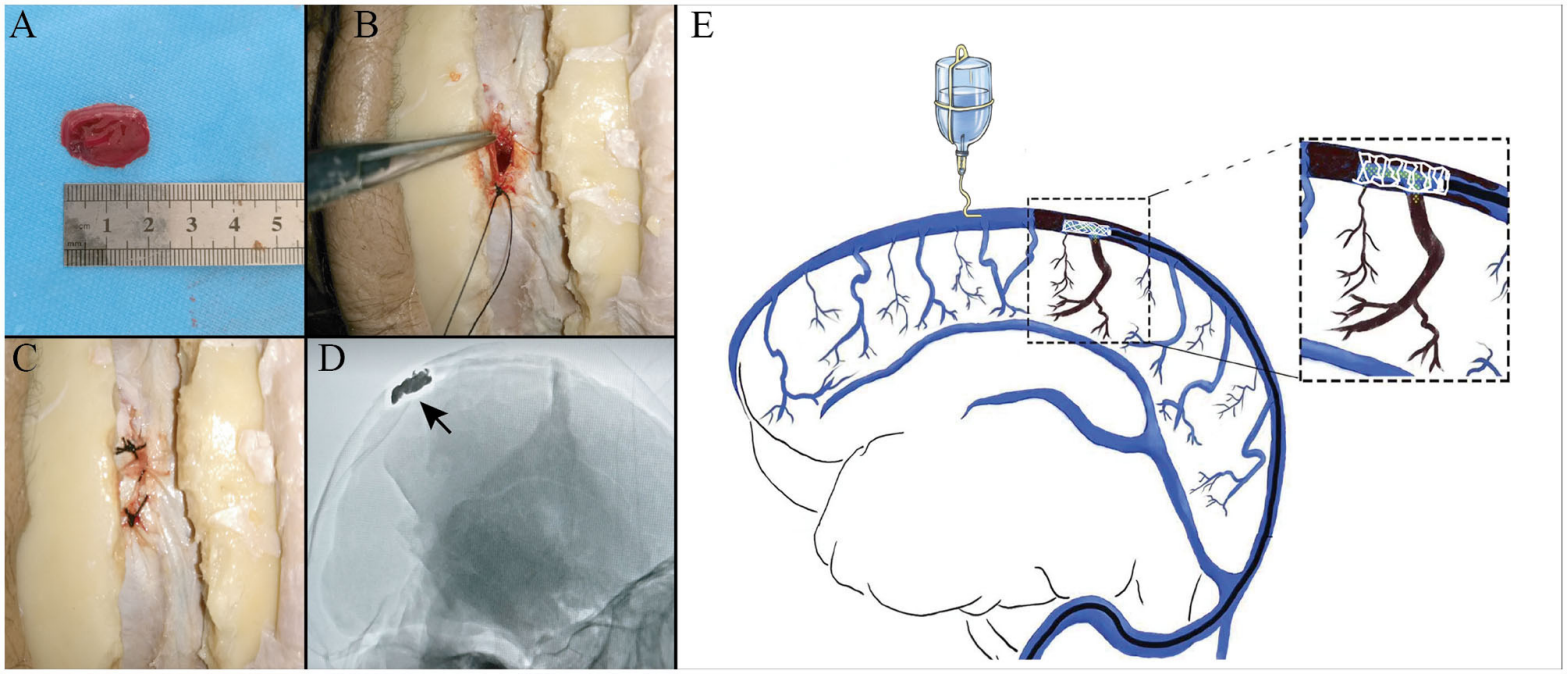
Experimental thrombus model in the superior sagittal sinus (SSS). Photographs showing that a thrombin-induced thrombus **(A)** was put into the sinus **(B)**, sutured in with surgical silk thread **(C)**, and confirmed by digital subtraction angiography (DSA) **(D)**. An illustration of mimicking mechanical thrombectomy in case of simulate normal blood flow **(E)**.

The scalp was removed with particular attention to expose the coronal suture and the inion. The calvaria around the coronal suture was removed with a power tool. The occipital bone around the inion was removed using a rongeur until the confluence of sinuses and the bilateral transverse sinuses were completely exposed. In order to simulate the normal blood flow in the SSS, a needle was inserted in the SSS anterior to the coronary suture, connected to a power injector, and a flow of saline was maintained at 10 cm/s under ultrasound monitoring (Brain Medicine, Inc., Nanjing, China). The SSS was opened superiorly at a region posterior to the coronary suture, and the 20 mm artificial thrombus was put into the sinus, which was then closed with a silk surgical suture. Once the saline outflow dwindled to a stop, bilateral transverse sinus was ligated with 4-0 suture (Figure 1).

### Mimicking Mechanical Thrombectomy in the Superior Sagittal Sinus

A 6-F Envoy guide catheter (Cordis, Inc., Miami, FL, USA) was placed at the transition from transverse to sigmoid sinuses via the left or right posterior mastoid bone window. A micro guidewire and a microcatheter were advanced retrogradely into the SSS, through the artificial thrombus, and stopped rostral to it. After the position of the catheter was confirmed by the poster–anterior and lateral projection digital subtraction angiography (DSA), the tip of the micro guidewire was kept in the anterior third of the SSS. The micro catheter was pulled out and replaced by a 4 × 20-mm Trevo Pro Vue stent retriever reaching to the thrombus along the micro guidewire. The stent was slowly pulled back to the transverse sinus. These processes were repeated three times on each cadaver sample and named as experiments 1, 2, and 3. Each experiment means that the stent was slowly pulled back to the transverse sinus.

### Thrombus Particle Analysis

Once the stent was pulled back to the transverse sinus, in order to collect the emboli, a saline flush from bilateral transverse sinus with a flow rate of 3.8 ml/s was used, and the total flush volume was 200 ml. The potential residual particles were identified under DSA and endoscope from confluence of sinuses. The emboli generated during the procedure were collected and measured with a caliper. The residual thrombus area in the SSS was observed via X-ray and calculated by Image J software.

### Assessment of Damage to the Chordae Willisii by Endoscope

Damage to the CW was evaluated using an endoscope, which was inserted into the lumen of SSS from the confluence of sinuses. Based on the degree of damage to the CW, the following classification was used in this study: Minor damage—tear on the surface of the CW; Serious damage—completely torn or split of the CW.

### Statistics

All statistical analyses were performed using *SPSS* 22 for Windows (IBM Corporation, Armok, NY, USA). Categorical data, including the percentage of large particles, were summarized using descriptive statistics. The numerical data were summarized as means ± SDs. One-way ANOVA followed by least significant difference (LSD) *post-hoc* was used to compare the number of particles and residual thrombus area in different procedures. The non-parametric chi-square analysis was also used to compare the large particles and damage to the CW between different procedures. A *p* < 0.05 was considered statistically significant.

## Results

### Number and Size of Visible Particles in the Mechanical Thrombectomy

**Figure 2:**
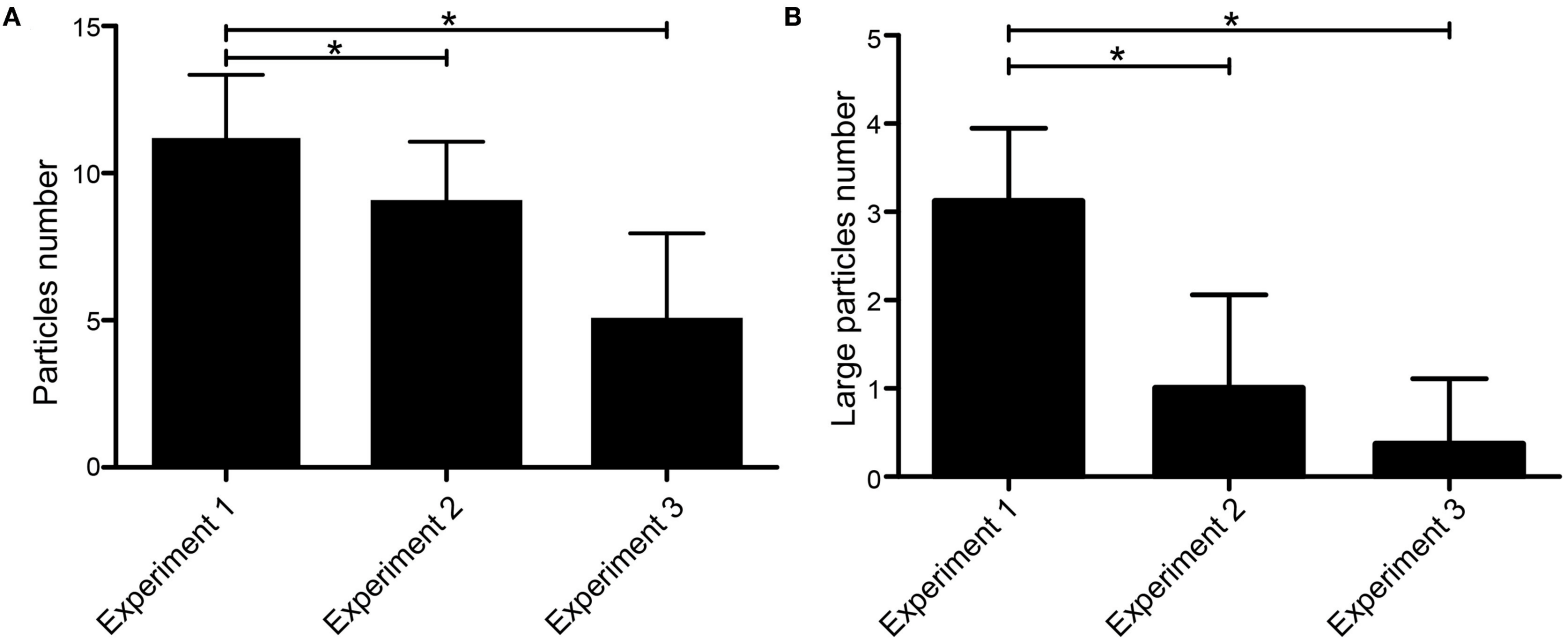
Number and size of visible particles in the MT. **(A)** Average number of emboli with size ≥1 mm produced by each experimental procedure. **(B)** Average number of large emboli with size ≥5 mm produced by each experimental procedure. *p < 0.05.

The average numbers of particles in experiments 1, 2, and 3 were 11.17 ± 2.17, 9.00 ± 2.07, and 5.00 ± 2.96, respectively. There was a significant difference in the number of particles between each experiment (*p* = 0.00) (Figure 2A). According to the diameter of thrombus particles, it can be divided into large (≥5 mm) and small particles (1–5 mm). The average rate of large particles in experiments 1, 2, and 3 were 29.06, 13.11, and 7.69%, respectively. The number of large particles produced by experiment 1 was significantly higher than the other two experiments (Figure 2B).

In case of simulate normal blood flow, in comparison with previously published data acquired under middle cerebral artery occlusion (19), the MT in the SSS produced more large particles than that in the cerebral artery occlusion (*P* < 0.05).

### Comparison of Residual Thrombus Area After the Mechanical Thrombectomy

**Figure 3:**
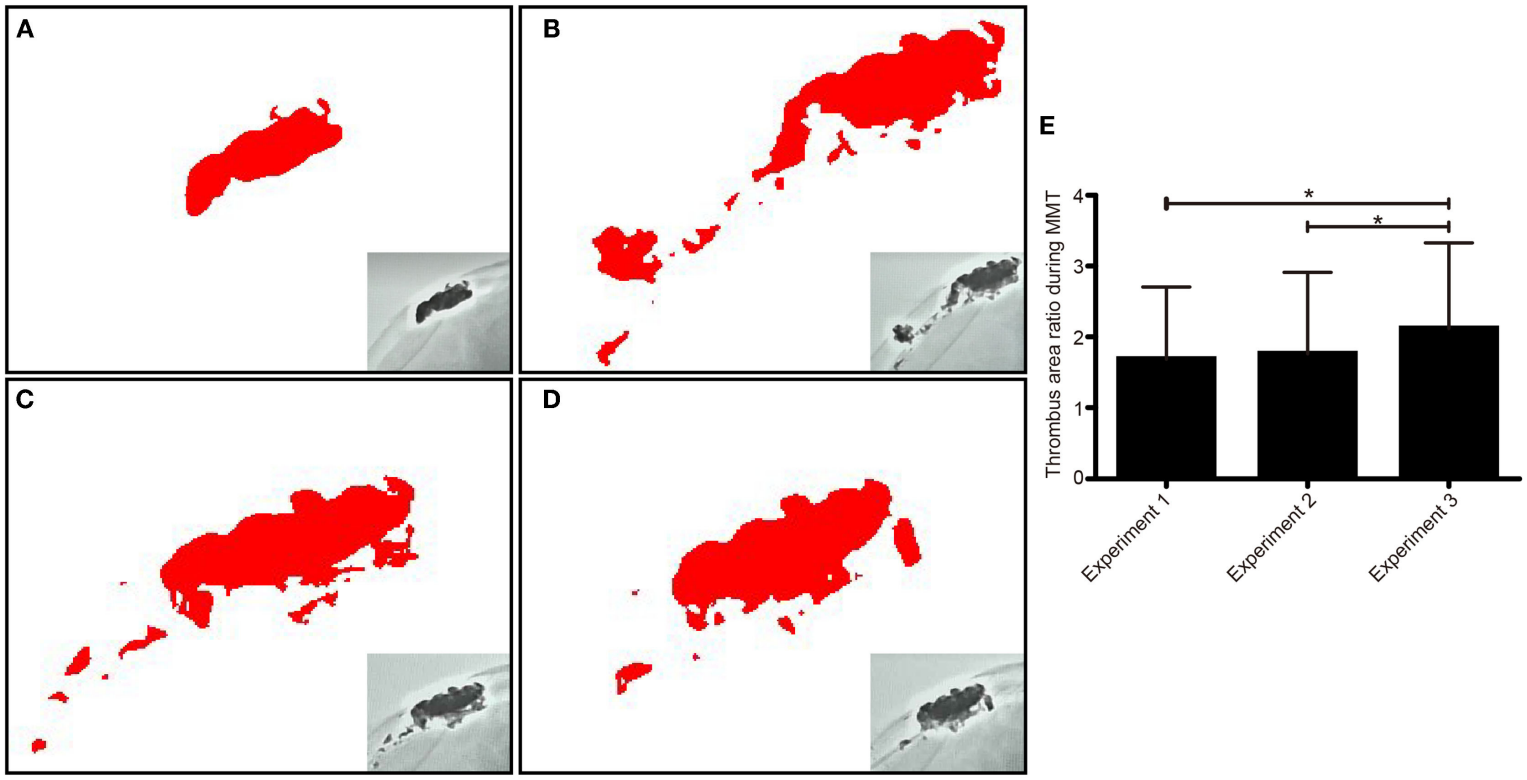
The residual thrombus area was assessed via X-ray and analyzed by image J software in each experiment. The thrombus morphology was intact before operation **(A)**. The thrombus morphology changed after experiment 1, which showed that a large particle separated from the thrombus **(B)**. The residual thrombus area was revealed after experiment 1 **(C)** and experiment 2 **(D)**. Comparison of residual thrombus area in repeated experimental procedures **(E)**. *p < 0.05.

The residual thrombus area was evaluated by ImageJ software. The average areas of the residual thrombus in experiments 1, 2, and 3 were 10,588.03 ± 6.93, 11,057.53 ± 7.43, and 13,325.45 ± 5.89 mm^3^, respectively. There was a significant difference in the thrombus area ratio before and after operation in experiments 1, 2, and 3 (*p* = 0.00) (Figure 3).

### Effect of Stent Damages to Chordae Willisii Observed by Endoscopy

**Figure 4:**
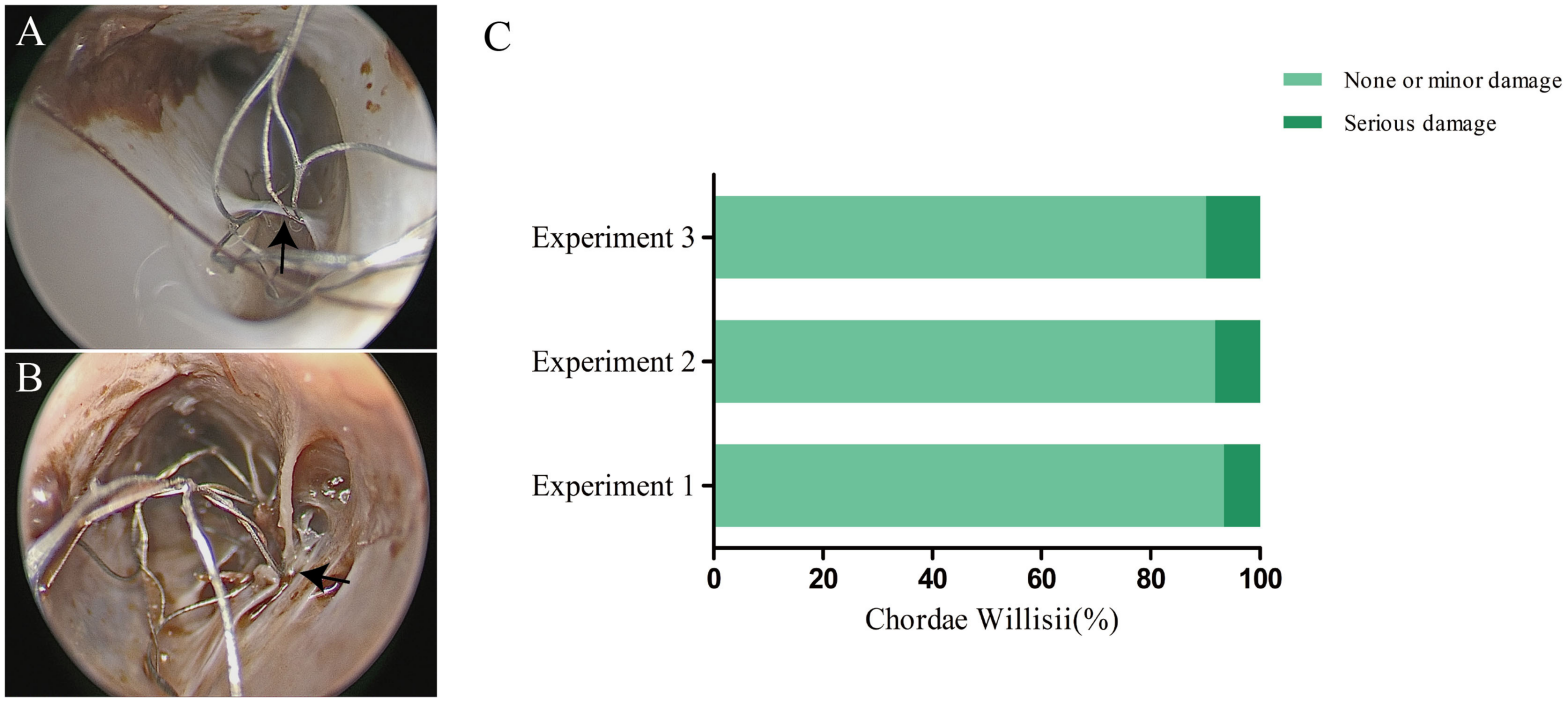
Effect of a stent on chordae willisii (CW) during repeated mechanical thrombectomy (MT) procedures. The minor **(A)** or serious **(B)** damage was caused by stent (indicated by arrow). Statistical analysis of damage to CW among three replicate experiments **(C)**.

There were 263 CWs in 16 examined sinuses, with a mean of 16.4 chordae per sinus. Based on our observation, damage to CW in the SSS happened at two locations, the CW surface and the junction between the inferior and lateral walls. The number of minor damage cases were 55 (90.16%) and serious damage cases 6 (9.84%). Among the minor damage cases, 49 happened on the surface, and 6 at the junction. Among the serious damage cases, five happened on the surface and one at the junction. The use of stent resulted in no significant increase in damage to CW during the three replicated procedures (Figure 4).

## Discussion

In this study, we established an SSS thrombus model-simulated MT in the SSS with three procedures on each sample and assessed thrombus particles produced by the procedures. We found that the first procedure produced more large particles than the other ones, and repeated procedures increased residual thrombus area. In addition, the stent could lead to minor damages to the CW, although repeated experiments did not increase the rate of damage. To the best of our knowledge, no data thus far are available in the literature that has described the impact on the thrombus and CW during MT either *in vitro* or *in vivo*.

### Stent Retriever Mechanical Thrombectomy in the Superior Sagittal Sinus

MT has become a very effective treatment for serious cerebral venous sinus thrombosis (26). MT destroys and cuts off the thrombus through physical force, makes the thrombus loose in the venous sinus, thus recanalizes sinus blood flow. It was especially suitable for patients with refractory CVST such as (I) long duration of thrombosis; (II) ineffective anticoagulant therapy alone or complicated with intracranial hemorrhage; and (III) clinical application of fibrinolytic drugs was impossible, or the dosage of thrombolytic agents has to be reduced after mechanical thrombectomy. Dashti et al. used the Solitaire AB) to treat cerebral venous sinus thrombosis. The results showed that the MT could improve the patient's clinical symptoms significantly without obvious complications (12). Radoslav et al. reported that patients with severe cerebral venous sinus thrombosis and cerebral hemorrhage were treated with aspiration and solitaire stent thrombectomy. They believed that aspiration and stent thrombectomy can effectively reconstruct the venous sinus blood flow as soon as possible, reduce the pressure in the sinus, and relieve the clinical symptoms (27).

High recanalization grade of CVT independently predicts good neurological outcome (28). In order to improve the recanalization rate of CVT, a common practice has been placing a microcatheter near the thrombus and infusing thrombolytic agents. However, only 40–50% of patients had partial or complete recanalization, and 7% had recurrence of thrombosis. MT combined with contact thrombolysis improves the rate of recanalization. Qureshi analyzed the data of patients with intractable CVS. Their results showed that 40% of the patients had complete recanalization; no new hematoma was found in the process of thrombolytic agents (10).

### Appreciation About Mechanical Thrombectomy in the Superior Sagittal Sinus

According to the traditional concept, there were arachnoid granules and fibrous cords in the cerebral venous sinuses (29). These structures were easy to be damaged during MT and lead to new thrombosis in the sinuses (22). When the microcatheter was placed to inject urokinase, one should try to avoid increasing the tension in the bridging vein. Thrombus particles formed during thrombectomy may cause pulmonary embolism. Ava Liberman et al. reviewed the occurrence of pulmonary embolism in patients with cerebral venous sinus thrombosis. Their results showed that the incidence of pulmonary embolism was 1.4%, far lower than that of pulmonary embolism caused by deep vein thrombosis. The main reasons could be that the diameter of the cerebral venous sinus was smaller than that of the deep vein in the lower extremity, and the volume of the thrombus in the sinus was smaller (30). In this study, we found that the diameter of thrombus particles during MT was mostly <5 mm, and the possibility of pulmonary embolism should be low. However, after thrombectomy, the loosen thrombus may lead to a change in thrombus morphology and may further develop into large thrombi particles, especially in the process of thrombolysis. As a result, this may lead to pulmonary embolism. In addition, our study found that there are still many thrombi residues in the sinus even after repeated MT procedures, which suggests that multiple approaches (thrombectomy, thrombolysis, and anticoagulation) should be combined to treat cerebral venous sinus thrombosis.

Moreover, the frequent passage of a stent through dura sinuses can sometimes cause idiopathic chronic intracranial hypertension due to sinus thromboses or hypertrophic arachnoid granulations. The results of this study showed that frequent usage of such kind of procedure may create minor iatrogenic damages to the lamellae and trabeculae and, therefore, may result in the exposure of collagen fibers in small areas. Clinically, intraoperative application of anticoagulant drugs could reduce the chance of thrombosis after MT.

### Limitations

We acknowledge that our study has some limitations. First, cadaveric vasculature and artificial thrombus may not truly reflect the flexibility of intracranial vessels and their interaction with the occlusive clot. Second, we used only a single type of clot that may not reflect the wide range of clots encountered in clinical practice. Third, the vasculature in a cadaver might differ and could have an impact in the results of the study. Finally, it was uncertain whether the emboli size could produce greater clinical complications. However, it is very likely that the emboli burden can impair patient outcome after MT. Further studies overcoming these limitations perhaps in a large sample size would show additional validation.

## Conclusion

MT could produce a large amount of thrombus particles and a minor damage to CW. Thrombus residues after MT suggests that postoperative anticoagulation therapy or prolonged microcatheter-based local thrombolytic infusion should be considered.

## Data Availability Statement

The raw data supporting the conclusions of this article will be made available by the authors, without undue reservation.

## Ethics Statement

The studies involving human participants were reviewed and approved by Ethics committee of the Guangxi Medical University. The patients/participants provided their written informed consent to participate in this study.

## Author Contributions

JY, YY, and JD conceived the study and designed the experiments. YY and JD conducted the experiments. YY, ZL, and SL analyzed and interpreted the data. YY drafted the manuscript. YY and ZL did the statistical analysis. JY and JH supervised the study. All authors contributed to the article and approved the submitted version.

## Conflict of Interest

The authors declare that the research was conducted in the absence of any commercial or financial relationships that could be construed as a potential conflict of interest.
